# Electrospray Deposition of PEDOT:PSS on Carbon Yarn Electrodes for Solid-State Flexible Supercapacitors

**DOI:** 10.1021/acsami.3c03903

**Published:** 2023-06-19

**Authors:** Mariana P. Moniz, Amjid Rafique, João Carmo, J. P. Oliveira, Ana Marques, Isabel M. M. Ferreira, Ana Catarina Baptista

**Affiliations:** † CENIMAT|i3N, Department of Materials Science, School of Science and Technology, NOVA University of Lisbon, 2829-516 Caparica, Portugal; ‡ Physics Department, Faculty of Sciences, University of Lisbon, 1749-016 Lisbon, Portugal

**Keywords:** electrospray, PEDOT:PSS, flexible supercapacitors, gel-polymer electrolyte, electronic textiles

## Abstract

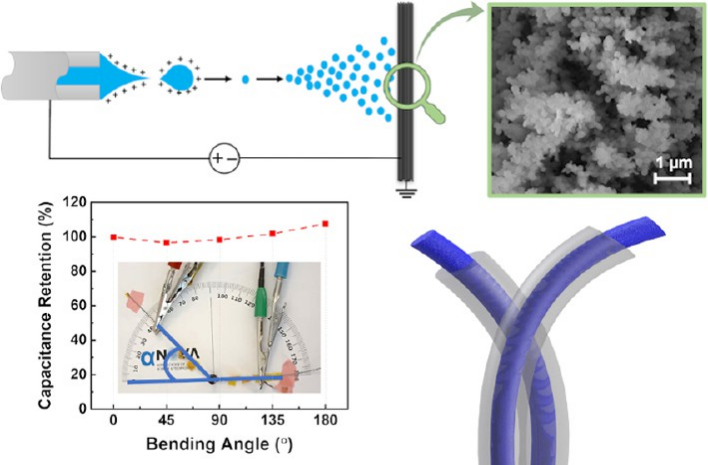

The increasing demand for flexible electronic devices has risen due to the high interest in electronic textiles (e-textiles). Consequently, the urge to power e-textiles has sparked enormous interest in flexible energy storage devices. One-dimensional (1D) configuration supercapacitors are the most promising technology for textile applications, but often their production involves complex synthesis techniques and expensive materials. This work unveils the use of the novel electrospray deposition (ESD) technique for the deposition of poly(3,4-ethylenedioxythiophene)–poly(styrene sulfonate) (PEDOT:PSS). This deposition methodology on conductive carbon yarns creates flexible electrodes with a high surface area. The deposition conditions of PEDOT:PSS were optimized, and their influence on the electrochemical performance of a 1D symmetric supercapacitor with a cellulose-based gel as an electrolyte and a separator was evaluated. The tests herein reported show that these capacitors exhibited a high specific capacitance of 72 mF g^–1^, an excellent cyclability of more than 85% capacitance retention after 1500 cycles, and an outstanding capability of bending.

## Introduction

Smart fabrics and e-textiles have been attracting tremendous attention^1^ due to the possibility of integrating flexible, lightweight, and comfortable microdevices in electronic textiles that will enable a technological breakthrough and will undoubtedly boost the transmission of flexible wearable electronics in our daily life.^2^ Flexible electronic devices such as communication systems and biomedical devices and multipurpose sensors have already been integrated into textiles.^3,4^ However, they lack a power supply that is compatible with such integration. In this regard, fiber-based capacitors, in one-dimensional (1D) configuration, that ensure flexibility and wearability and can be easily weaved in conventional textiles are required. Flexible supercapacitors (FSCs) and batteries are potential contenders for power supplies, but the environmental susceptibility of the latter, and the usage of toxic chemicals as electrolytes are subjects of concern.^5,6^ FSCs’ key challenge is the design and fabrication of active materials with robust mechanical flexibility and high energy coupled with remarkable cyclic and bending stability.^7,8^

Active materials for FSCs range from carbonaceous materials (e.g., CNT,^9^ graphene,^10^ and carbon nanofibers^11^), to transition metal oxides/dichalcogenides (e.g., MnO_2_^12,13^ MoS_2,_^13^ RuO_2,_^14^ NiO_2,_^15^ Co_3_O_4_^16^), conducting polymers (e.g., polyaniline (PANI),^17^ polypyrrole (Ppy),^18^ polythiophene^19^) and composite materials (e.g., rGO/Fe_2_O_3_^20^ and PANI/MnO_2_^21^). In a generic designation, supercapacitors (SCs) are classified into two main categories based on their charge storage mechanism: (i) electric double-layer capacitors (EDLCs), in which charges are stored on the surface of the active material and hence capacitance and energy density values are surface area-dependent, and (ii) pseudocapacitors (PCs) that combine surface area and faradaic reactions and have a 10-fold higher specific capacitance and energy density than EDLCs.^22^

Among the existing pseudocapacitive materials, conducting polymers exhibit excellent electrical conductivity compared to transition metal oxides; therefore, they are considered as one of the promising electrode materials for high-performance FSCs.^23^ Poly(3,4-ethylenedioxythiophene) (PEDOT), as a polythiophene derivative, is an environmentally friendly, stable, and easy-to-process material compared to other conducting polymers, and it possesses good electrical conductivity and excellent electrochemical performance.^24^ However, since PEDOT is insoluble in water and in some organic solvents, its applicability is restricted in different fields. Polystyrene sulfonate (PSS) on the PEDOT backbone^25^ resulted in a polymer (PEDOT:PSS) that is soluble in water and other organic solvents, facilitating its solution processability. Due to the high electrical conductivity, optical properties, and easy processing of PEDOT:PSS, its application in different flexible electronic devices such as light emitting diodes (LEDs),^26^ solar cells,^27^ and supercapacitors^28^ has been widely exploited. Also, the use of PEDOT:PSS as a coating, thin film, or active material has been widely reported.^29^ For instance, Du et al. prepared a PEDOT:PSS-based electrode with deposition on cellulose nanofibril papers for FSCs with an areal specific capacitance of 854.4 mF cm^–2^ and an areal energy density of 30.86 μWh cm^–2^.^19^ Khasim et al.^30^ developed a high-performance FSC using rGO/PEDOT:PSS nanocomposites by secondary doping, obtaining active materials and devices with a specific capacitance of 174 Fg^–1^ and 810 Wh kg^–1^, which is 4 times higher than that of pristine PEDOT:PSS, exploring the synergic effect of carbon and conducting polymers. Yet, SCs using PEDOT:PSS without any packaging cannot sustain severe mechanical stresses during the electrochemical evaluations owing to poor adherence.^23^ According to the literature, the deposition of conducting polymers on flexible substrates comprising nanofibers can be an effective approach to enhance the conductivity of the substrate, but it depends on the deposition techniques used.^31^

Several techniques have been reported for PEDOT:PSS coatings on textile-based substrates such as dip coating,^32^ soaking,^33^ dipping,^34^ drop casting,^35^ immersing,^36^ inkjet printing,^37^ spin coating,^38^ and spray coating.^39^ The major limitations of those coatings are the weak adhesion to substrates,^18^ sensitivity to solvent evaporation, irregularity in the distribution of the material due to gravity-induced drainage, and wastage of the solution, while a large quantity of solution needs to be used for creating very thin layers of material.

Electrospray (ES) has been reported as a technique for the functionalization of flexible substrates that is compatible with different solvents for device fabrication.^40^ During the electrospray deposition (ESD) technique, the balance between an electrostatic force and the solvent surface tension generates one or multiple charged, monodispersed droplets.^41^ Before starting the ESD, the active material is diluted within a suitable solvent, and the resultant solution is pumped through a needle connected to a high-voltage source. During electrospray, at the tip of the nozzle, the charged active material forms a “Taylor cone” and generates microscale droplets at the high-field zenith. During the flight of the droplet to the target substrate, the solvents evaporate, and the size of the droplet shrinks to the “Rayleigh limit,” where surface charges overcome the surface tension causing the droplet to face Coulomb fission and forming child droplets. The formation of these small droplets creates a spray plume that can uniformly cover large surfaces with minimal use of material, creating large surface areas.^41^ ESD offers several advantages compared to its counterparts (traditional deposition techniques), including (i) the formation of monodisperse droplets and highly uniform deposition; (ii) the formation of micro/nanosized droplets, which makes it an effective technique for micro/nanoscale coatings; (iii) easy parameter control to obtain desired morphologies (namely, flow rate, applied potential, and tip-to-target distance); (iv) the effective use of an active material, meaning that a small quantity of material is wasted using this technique; and (v) the possibility of deposition in ambient conditions. These advantages have led to the application of ESD for the fabrication of thin films based on nanoparticles^42^ or conductive polymer coatings.^43^ Electrospray is a widespread technique in the development of materials for flat energy applications. However, to the best of our knowledge, this work reports for the first time the use of electrospray to functionalize 1D substrates (or yarns) as electrodes for supercapacitors.

Herein, carbon yarns were coated with PEDOT:PSS by ESD, and their application as electrodes in solid-state FSCs was studied. The coating was optimized by changing the processing parameters. These electrodes, coupled with the application of an all-solid-state electrolyte made of cellulose acetate, allowed the production of fiber-shaped supercapacitors with high flexibility, conformability, and excellent bending stability.

## Experimental Section

### Materials

All chemicals of analytical grades were used in the experiments as received without further purification. The full list is as follows: commercial carbon yarn (TenaxTM-E HTA40 E13 3K200tex, 1.6 × 10^–3^ Ω·cm), PEDOT:PSS (Clevios (Ossila) TM Al4083, Heraeus), poly(vinylidene fluoride) (PVDF) membrane filters 0.45 μm (FilterBio), isopropanol (IPA) (Sigma-Aldrich, purity ≥99.5%), acetone (Honeywell, purity ≥99.5%), cellulose acetate (CA) (Sigma-Aldrich, average *M_n_* ∼ 50,000), potassium chloride (KCl) (Sigma-Aldrich, purity 99.5–100.5%), and poly(ethylene glycol) (PEG-200) (Sigma-Aldrich, average mol wt 200).

### Material Preparation

Before functionalization of the carbon yarns (CYs), they were cleaned with acetone to eliminate any surface coatings or impurities. The CY was placed inside an acetone solution for 40 min and after that time, CYs were placed on a heating plate at 90 °C for 1 h to completely dry them. Just before deposition, the CY was placed under ultraviolet (UV) light for 30 min to improve its wettability and eliminate any remaining organic impurities.

The PEDOT:PSS electrospray solution was made with pristine PEDOT:PSS filtered through a 0.45 μm PVDF filter and then diluted in an IPA solution and deionized water (DI water) in a volume proportion of 4:6:1 of PEDOT:PSS, IPA, and DI water respectively. The solution is stirred for 8 h and then stored in the fridge.

A CA-based gel-polymer electrolyte was prepared; for 100 mL solution, 7.5 g of CA was mixed with 75 mL of acetone until complete dissolution occurred. Then, 25 mL of PEG-200 was added to 5 g of KCl, followed by stirring until a homogeneous dispersion of PEG was formed.

### Electrospray Deposition of PEDOT:PSS

The electrospray deposition setup consists of a 1 mL syringe (B. Braun) connected to a metallic point needle with gauge 23 (ITEC), a syringe pump (New Era Pump Systems, Inc, NE-300), and the CY substrate in a multicoater machine (LRC multicoater). A nonconductive frame was used to fix the grounded CY and stretch it fully. In this setup, the needle and syringe are horizontally aligned with the substrate (as shown in Figure S1). The electrospray deposition parameters studied were the flow rate between 40 and 80 μL h^–1^, applied voltage between 12 and 18 kV, needle tip-to-collector distance between 4 and 10 cm, and deposition times of 5, 10, 20, 30, and 60 min. The deposition time for coating the fibers corresponds to PEDOT:PSS deposition of one side of the carbon yarns. For total coverage of the fibers, the deposition is repeated under the same conditions and in the same time span on the other side of the fibers. This is achieved by rotating the fiber carrier substrate by 180 degrees and repeating the deposition in the same conditions. Temperature and relative humidity inside the deposition chamber were kept constant in value intervals between 18-25 °C and 42-48%, respectively.

### Assembly of Devices

Both electrodes of the supercapacitor were dipped into the gel-polymer electrolyte and twisted to assemble a symmetrical device. The electrolyte solution coverage was formed by successive periods of 5 s dips in the solution and dried in ambient conditions for 15 min. This cycle resulted from a preliminary study being the one leading to the minimum thickness required to electrically isolate the entire yarn. Then, the two electrolyte-coated carbon yarns were manually coiled around each other, with the number of turns fixed at 6 to ensure reproducibility and comparability among devices.

### Characterization

The morphology of the samples was analyzed by an optical microscopy (OM) (Leica DMi8) system and a scanning electron microscopy (SEM) (Carl Zeiss Auriga crossbeam SEM-FIB workstation instrument equipped with an Oxford Instruments Aztec X-ray energy-dispersive spectrometer). The chemical composition of the CYs and the active material was investigated by Raman spectroscopy (Witec alpha300 RAS) using an excitation wavelength of 532 nm. The laser power was set to 0.6 mW, and all spectra were acquired for 5 min; in the case of the bare carbon yarn Raman spectrum, this was obtained for 5 min, but with a laser power of 1.6 mW.

The capacitive behavior of the CY and PEDOT:PSS-functionalized carbon fibers was determined by cyclic voltammetry (CV), galvanostatic charge and discharge cycling (CD), and electrochemical impedance spectroscopy (EIS) measurements. These measurements were performed with a Gamry 1010 Potentiostat/Galvanostat.

### Calculation of the Specific Capacitance

Electrochemical characterizations were performed to evaluate the electrochemical performance of the supercapacitors. The experiments were performed in a two-electrode configuration. CV curves were recorded in a voltage window ranging from 0 to 1 V at different scan rates (5, 30, 50, and 100 mV s^–1^, respectively). Capacitance was calculated with eq 1 using CV plots.^22^
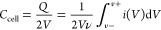
1where *C*_cell_ is the capacitance of the cell, *i* is the current, ν is the scan rate, and *V* (*V* = *V*_+_ – *V*_–_) describes the potential window. Mass specific capacitance was determined using eq 2, which was used for calculating the mass of a whole electrode (PEDOT:PSS/CY).
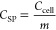
2where *m* denotes the mass of the electrodes.

## Results and Discussion

**Figure 1:**
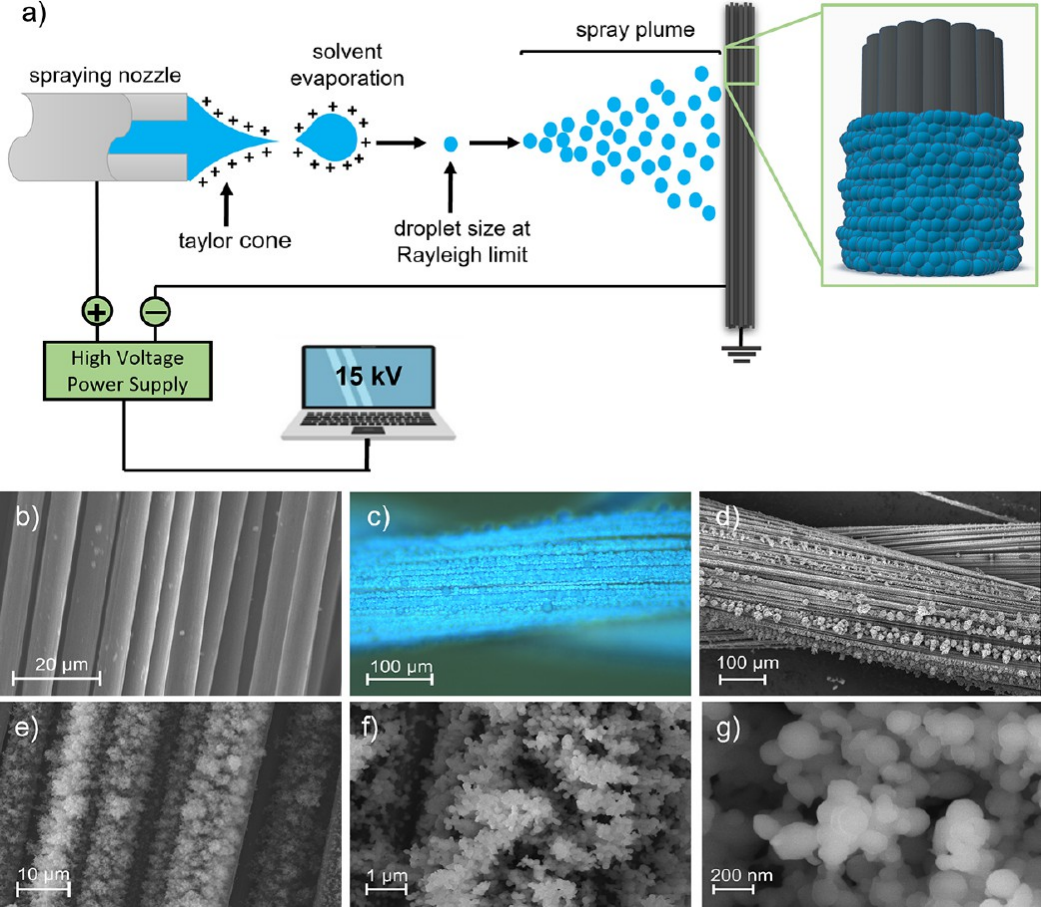
(a) Schematic representation of the e-spray deposition of PEDOT:PSS on the carbon yarns; (b) SEM image of the pristine carbon yarn; (c) optical microscopy image of the PEDOT:PSS-deposited carbon yarn; and (d–g) SEM images of PEDOT:PSS-functionalized carbon yarn at different magnifications.

The electrospray jet can work in two modes based on the ejection of the solution: continuous ejection mode and drop-on-demand ejection mode.^44^ In the continuous ejection mode, the jet appears from the tip of the syringe and collapses in the stream of the solution, while in the drop-on-demand mode, droplets emerge from the cavity of the syringe. Under the applied potential and effect of numerous parameters, these modes can be exploited to achieve three different patterns: dots, lines, and thin films.^45^ The most attractive advantages of ESD to this work include being an extremely conformable technique, meaning that it can deposit highly uniform thin films on diverse shapes of substrates (wires and flat surfaces, for example) and that it demands a very low amount of material, which is advantageous especially when working with expensive materials.^46^ ESD is a deposition technique that creates a spray-type deposition without the need for a pressured gas based on the principle of electromagnetic attraction. Due to a high applied potential, the jet disintegrates into charge monodispersed droplets, which go through a process of solvent evaporation. On connecting the target substrate to the ground, the charged droplets are attracted toward the target substrate, as schematically detailed in Figure 1a. In the process of being transported to the substrate, the solution forms a spray plume. The solvent fully (or partially) evaporates, and the droplet size decreases until reaching the Rayleigh limit, creating a thin film of PEDOT:PSS on the substrate.^47^ During ESD deposition, two factors are most critical: (i) achieving uniform PEDOT:PSS droplets by exploiting the electrospray mode of deposition and (ii) deposition of hydrophilic PEDOT:PSS thin films on the carbon yarn substrates by controlling the wettability of PEDOT:PSS droplets. Various techniques can be employed to reduce the surface tension of the PEDOT:PSS solution such as adding a surfactant to the spray solution or mixing secondary solvents with lower surface tension. In the solution preparation for ESD, IPA and DIW were added to PEDOT:PSS to reduce the surface tension and to prevent agglomeration of the PEDOT:PSS particles.^48^ Under an applied potential of 15 kV, with a fixed tip-to-target distance of 7 cm and a flow rate of 60 μL h^–1^, the electric field between the capillary tip and the target CY allows the solution to overcome the surface tension of the droplet and atomize into a spray plume. In the end, thin films were deposited by spreading the small droplets, which overlay and coalesce with the already deposited ones, creating a tightly packed, surface-covering, nanoparticle-based film, as represented in the schematics of Figure 1a.

The morphology of the pristine CYs and PEDOT:PSS-functionalized CY was analyzed by OM and SEM. Figure 1b shows the SEM image of the pristine CY, and Figure 1c,d shows, respectively, the OM and SEM images of the functionalized CY. The presence of PEDOT:PSS over the surface of the carbon yarn is clear from the change of color of the CY as well as the change in morphology, which is evident in the SEM images. The PEDOT:PSS coating has a spherical nanostructure-type morphology of both agglomerates and nanoparticles forming within these agglomerates, as depicted in the SEM images of Figure 1d–g. Moreover, SEM imaging confirms that the carbon yarn is coated with a continuous film of PEDOT:PSS. Figure 1f,g confirms the spherical-like morphology of the PEDOT:PSS nanoparticles and also the formation of a highly porous coating that preferentially covers the fibers of the yarn, leading to very good fiber coverage. During ESD, the electrically charged particles of PEDOT:PSS traveled from the syringe to the substrate, and after complete solvent evaporation, deposited as porous thin films with spherical-shaped nanostructures on the surface of the fibers of the carbon yarn.^49^ This type of porous structure and surface coverage created a high surface area, which is beneficial for the fabrication of PEDOT:PSS/CY composite electrodes as potential electrodes for application in FSCs.

**Figure 2:**
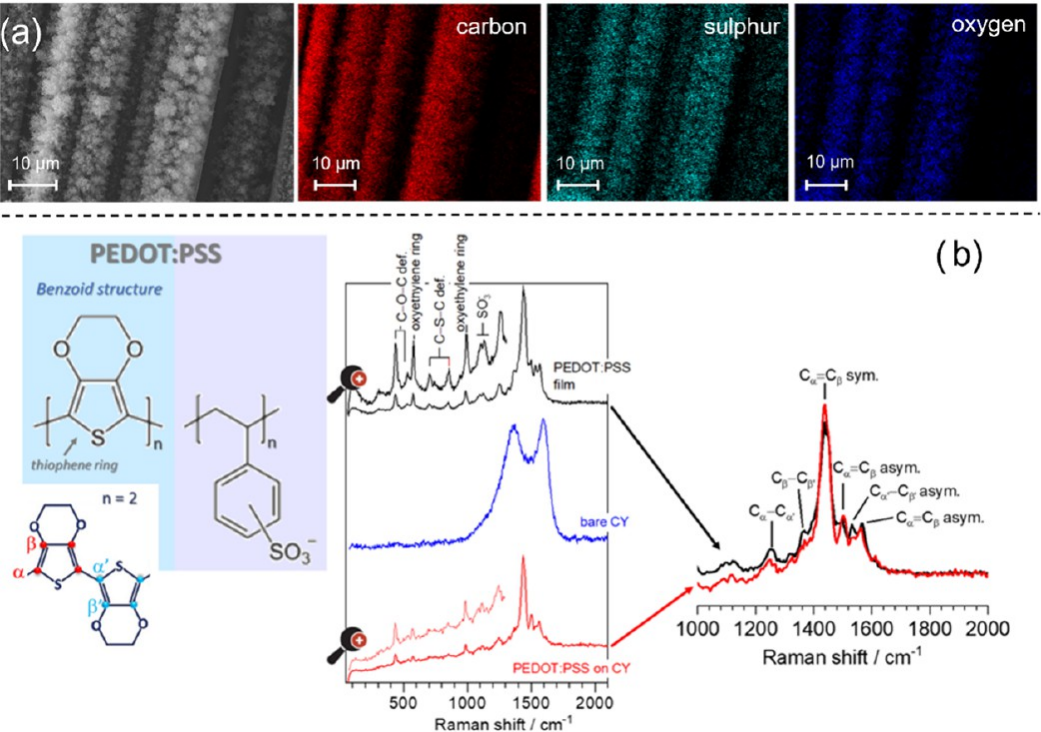
(a) SEM and EDX images of C, O, and S in the PEDOT:PSS-coated CY. (b) Raman spectra for ESD PEDOT:PSS deposited on the glass (top) and carbon yarn (bottom), corresponding to the Raman spectrum in blue to the pristine carbon yarn.

To confirm the PEDOT:PSS thin-film deposition, electron-dispersive X-ray spectroscopy (EDX) analysis was performed on the coated yarns, as shown in Figure 2a. EDX spectra confirm the elemental presence of constituent elements of PEDOT:PSS such as sulfur. Moreover, elemental mapping was also performed to identify the distribution of PEDOT:PSS nanoparticles present in the composite film. Carbon, oxygen, and sulfur were uniformly distributed throughout the composite film, which confirms the uniform distribution of the PEDOT:PSS film over the fibers.

The composition of pristine CYs and PEDOT:PSS-functionalized CYs was further analyzed by Raman spectroscopy, which allowed not only confirming the EDX results but also inferring the chemical structure that PEDOT:PSS formed after deposition. Figure 2b shows the Raman spectra of the raw materials and of the yarn coated with PEDOT:PSS under green light excitation with a wavelength of 532 nm. The film of PEDOT:PSS was first deposited on a flat glass to ensure good adherence and to allow subsequent comparison of its chemical composition and structure with those of a film deposited on the CY. The Raman spectra of the PEDOT:PSS films deposited on the glass and on the yarns are identical and reveal that their thickness is thick enough (laser penetration depth is 1 μm) to prevent the detection of any signal from the substrate (glass or CY). In the carbon yarn spectrum, the two characteristic D and G bands were detected at 1372 and 1592 cm^–1^, respectively. As previously reported, the G band is characteristic of the sp^2^ carbon atoms in the two-dimensional (2D) hexagonal lattice, and the D band is attributed to structural defects and disorder in the hexagonal lattice.^18^ For this wavenumber range (∼1000–2000 cm^–1^), both PEDOT:PSS films not only reveal a similar chemical composition as well as structure, since in their Raman spectra, the position of the prominent *C*_α_ = *C*_β_ symmetric stretching vibration peak is at 1438 cm^–1^. This mode is typically assigned to the PEDOT:PSS benzoid structure^50^ (coil conformation) schematically shown in Figure 2b. The main vibrational modes are listed in Table S1 of the Supporting Information, and identification was made according to the literature.^50,51^

### Optimization of Deposition Conditions

The voltage applied between the tip of the needle and the target, the needle tip-to-target distance, and the flow rate are the main ESD parameters influencing the thin-film uniformity and morphology. Therefore, their impact on the morphology of the coatings was systematically studied by performing several deposition runs, and in each run, one parameter was varied at a time while keeping the others unchanged.

#### Effect of the Working Distance

**Figure 3:**
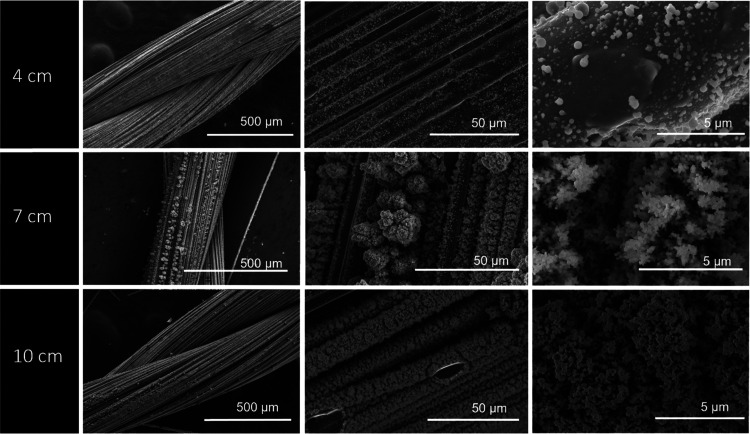
SEM images of electrospray-deposited PEDOT:PSS at different distances of 4, 7, and 10 cm, with constant parameters of an applied voltage of 15 KV, a flow rate of 60 μL h^–1^, and a deposition time of 30 min.

When an accelerated ES solution droplet moves toward the target, the time for solvent evaporation, until the solution reaches the target, is proportional to the needle tip-to-target distance. This distance also influences the electric field, as the electric field reduces with the increase of needle tip-to-target distance. The distance must be such that it can lead to an electric field strong enough to make the droplet reach the target and simultaneously let the solvent evaporate in that path, which also constrains the range of solvents available for the ES solution. Three distances were studied: 4, 7, and 10 cm. When the working distance is set to 4 cm, it is observed that PEDOT:PSS particles tended to fuse with other surrounding particles on the CY substrate (refer to Figure 3). This coalescence happens owing to incomplete evaporation of the solvent. The generation of uniform-sized spherical-shaped nanoparticles of PEDOT:PSS was difficult to achieve at this working distance. With the distance set to 7 cm, PEDOT:PSS particles became more uniform in size and in shape, as shown in Figure 3. According to the literature, until reaching a distance that allows the complete evaporation of the solvent, the particle size is highly dispersed and generally higher at smaller distances due to both incomplete solvent evaporation and particle coalescence.^52^ If the distance is too small, ESD may form a continuous film instead of a nanostructured one. On the other hand, at 10 cm, total evaporation of the solvent occurs, allowing the formation of a nanoparticle-based film. However, due to an increase in distance, the electric field is smaller, leading to fewer particles reaching the target, i.e., the carbon fibers. According to the literature,^52^ the working distance has to be optimized to ensure the complete evaporation of the residual solvent in the PEDOT:PSS nanoparticles and to avoid recombination and poor collection efficiency. On the other hand, after the point where the distance allows the complete solvent evaporation, the particle size increases with increasing distance. At smaller distances, there is a higher electric field that leads to the formation of smaller particles,^53^ and with these smaller particles, there is a formation of a film with a higher surface area. Based on this analysis, 7 cm was the selected distance for the deposition process since it created a coalesced particle type of film with a high surface area while allowing for complete solvent evaporation.

#### Effect of the Deposition Time

**Figure 4:**
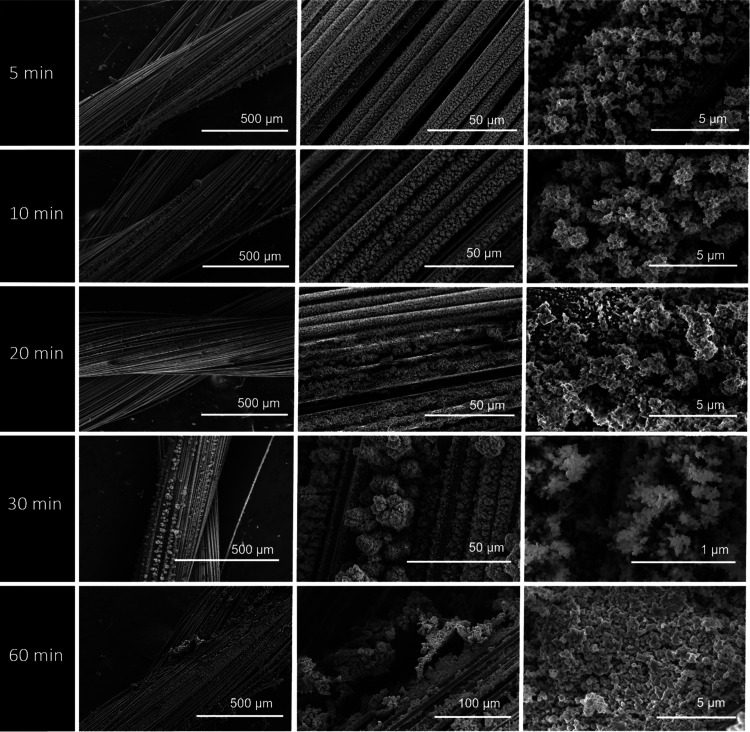
SEM images of electrospray-deposited PEDOT:PSS at different lengths of time of 5, 10, 20, 30, and 60 min, with constant parameters of a working distance of 7 cm, an applied voltage of 15 KV, and a flow rate of 60 μL h^–1^.

The effect of the electrospray deposition time on the morphology of the PEDOT:PSS nanoparticles was studied for different periods of time (5, 10, 20, 30, and 60 min), and SEM analysis was performed to analyze the influence on the morphology of the coatings. Figure 4 shows that deposition time has no direct influence on the morphology of PEDOT:PSS nanoparticles and only impacts the film thickness. As expected, the film thickness increases with the increase of deposition time.^54^ As compared to other deposition times, 60 min of deposition led to the formation of larger PEDOT:PSS nanoparticle aggregates, uniformly packed, producing a compact and thick film. Although there is no effect on the morphology of the deposited layers, the film thickness and mass loading will affect the electrochemical performance of the material and also the flexibility of the yarns. This behavior will be further discussed in the next section.

#### Effect of the Flow Rate

**Figure 5:**
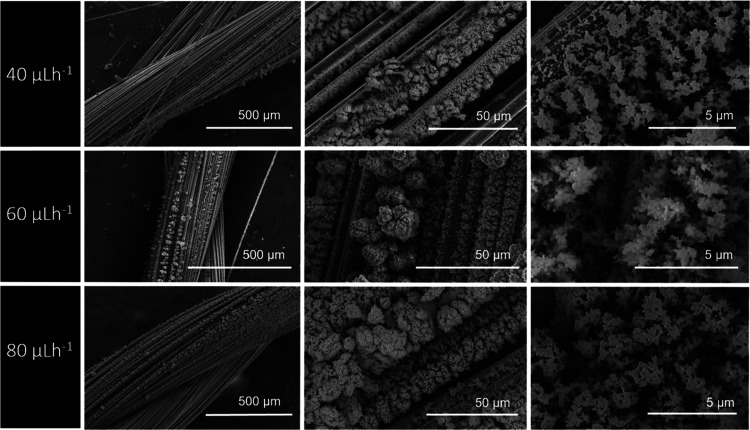
SEM images of electrospray-deposited PEDOT:PSS at different flow rates with constant parameters of a working distance of 7 cm, an applied voltage of 15 KV, and a deposition time of 30 min.

In the ESD process, a voltage-flow rate operating diagram is usually used to describe the cone-jet behavior.^55^ Normally, a steady cone-jet is formed in a certain stability window, and it is a well-established fact that increasing the flow rate affects the average size of the electrosprayed nanoparticle, which increases as the flow rate increases.^56^ In this study, three different flow rates were tested, 40, 60, and 80 μL h^–1^, and the effect of the flow rate on the coating morphology was studied. It is possible to observe that at a constant applied potential and specific working distance, the flow rate changes the size of the deposited particles, as shown in Figure 5. This also indicates that the particle size could be controlled more effectively by the flow rate in a prescribed stability window. On the other hand, it is also evident that at a lower flow rate, such as 40 μL h^–1^, CY threads were not fully covered for this specific time window due to low mass loads. Thus, an optimal flow rate level of 60 μL h^–1^ is required, which enables more control of the particle size and electrosprayed particle morphology within the selected time window.

#### Effect of the Applied Voltage

**Figure 6:**
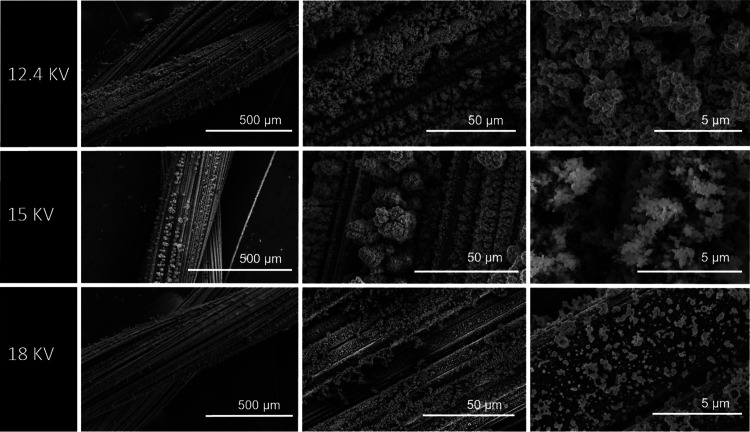
SEM images of electrospray-deposited PEDOT:PSS at different applied potentials of 12.4, 15, 18 kV, with constant parameters of a working distance of 7 cm, a flow rate of 60 μL h^–1^, and a deposition time of 30 min.

During ES deposition, sufficient voltage must be applied to overcome the surface tension of the droplet at the tip nozzle, and thus it controls the morphology and size of the electrosprayed PEDOT:PSS nanoparticles. It is generally accepted that an increase in applied voltage decreases the size of the deposited particles. However, optimum voltage is required to obtain a stable cone jet during ESD, which translates into monodispersed particles and better repeatability and reproducibility of the thin film and nanoparticles. These effects were studied for different applied voltages as this influenced the stability of the cone jet formed during ESD. For potentials lower than 12 kV, it was noticed that no deposition of PEDOT:PSS nanoparticles occurred. When the potential was increased to 12.4 kV, an unstable Taylor cone formed. This allowed the deposition of PEDOT:PSS but, as shown in Figure 6, without a consistent control of its uniformity, both in terms of coverage and morphology. At 15 kV, ESD generates a stable and constant Taylor cone creating uniform-sized spherical shape structures and individualized particle formation when compared with the deposition at 12.4 kV, as shown in Figure 6. Increasing the applied potential to 18 kV results in a film-like structure being obtained, similar to the one observed for the deposition using a working distance of 4 cm (as shown in Figures 3 and 6). This can be explained by the unstable spray associated with the application of high voltages that may lead to the acceleration and coalescence of the drops before reaching the target. Other works have reported that a high applied voltage can lead to polymeric solutions forming a continuous jet instead of individual particles.^57^ Considering the process stability at 15 kV, this potential was selected to produce a uniform coating of homogeneous PEDOT:PSS nanoparticle structures. At these conditions, with a needle tip-to-target of 7 cm, the electric field is 2.1 kV cm^–1^.

### Electrochemical Characterization

PEDOT:PSS-functionalized CYs were used both as flexible current collectors and active materials. Before analyzing the electrochemical properties of these electrodes, a cellulose acetate (CA)-based green gel-polymer electrolyte was prepared. Most gel-polymer electrolytes reported in the literature are enriched with acidic solvents such as H_3_PO_4_ and H_2_SO_4,_ which hinder their application in wearable supercapacitor applications, owing to safety issues, as these may cause skin irritation to the wearer.^58^ The electrolyte used, acting both as a separator and an ion mediator, is composed of CA, polyethylene glycol (PEG), and potassium chloride (KCl), all harmless to human health and easy to prepare.

**Figure 7:**
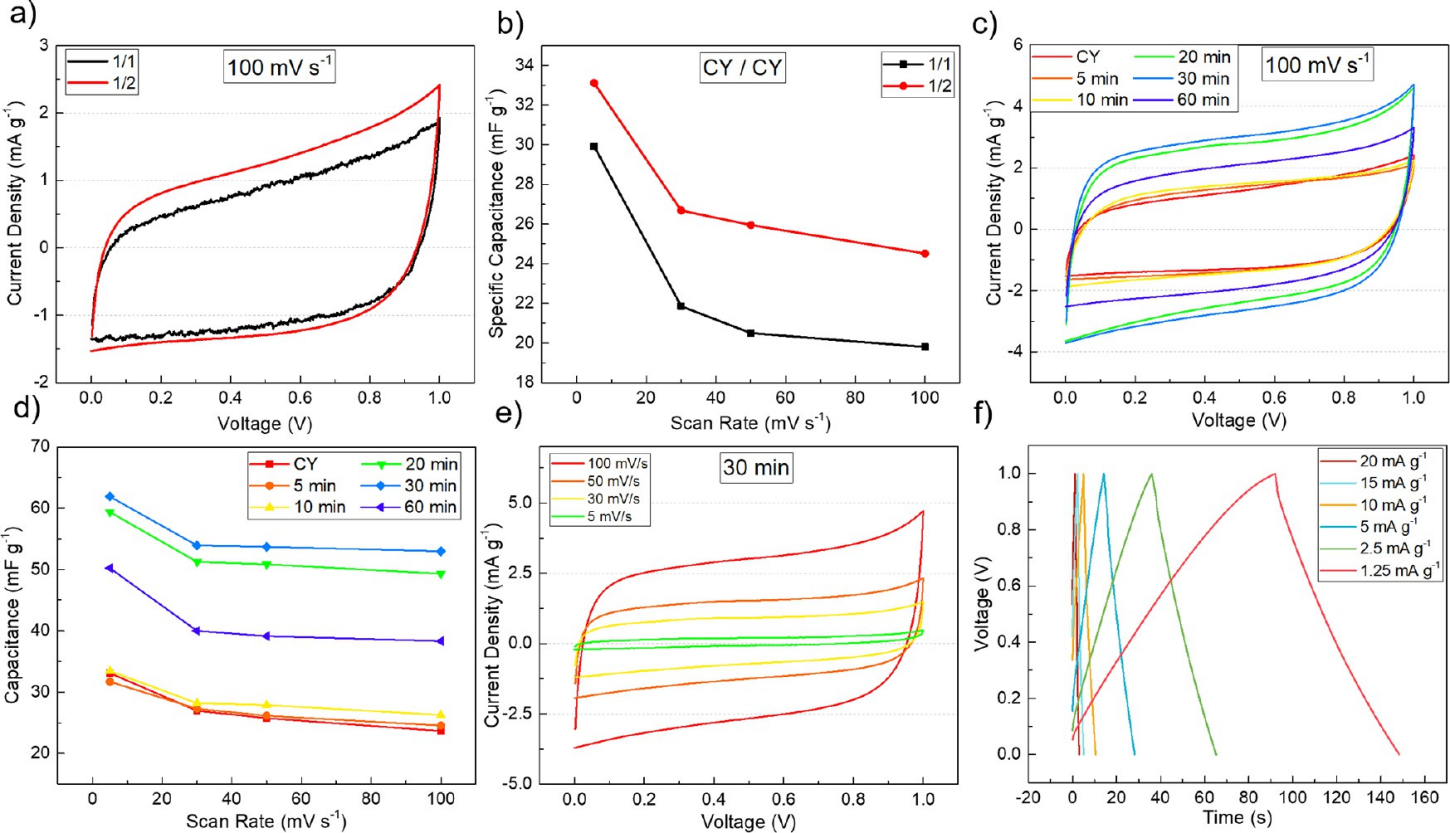
(a, b) Comparison of CV curves and corresponding specific capacitance for gel-polymer electrolyte coating optimization, (c) comparison of CV curves for electrospray deposition of PEDOT:PSS at different times of deposition, (d) comparison of specific capacitance at different times of deposition, (e) comparison of CV curves recorded for the best devices at 30 min of deposition, and (f) GCD curves for the 30 min device at different current densities.

Fiber-shaped supercapacitors were fabricated with pristine CYs as inner and outer electrodes in a twisted configuration with 6 turns, the standard number of turns around the inner electrode, to assess the best electrolyte configuration. Figure 7a,b shows the cyclic voltammograms and capacitance versus the scan rate of twisted electrode symmetric capacitors by varying the thickness of the electrolyte here defined as (1/1) one dip coating of electrolyte on each electrode and (1/2) one coating on the outer electrode and two coatings on the inner electrode. OM was used to estimate the thickness of the electrolyte layers, resulting in 96.61 ± 67.44 μm of electrolyte thickness for 1 dip and 296.04 ± 98.70 μm for 2 dips (the pictures used for these measurements are shown in Figure S2 of the Supporting Information). Figure 7a,b clearly shows that the device with the (1/1) electrolyte configuration shows a lower specific capacitance than the device with the (1/2) configuration. As the increase in thickness corresponds to a higher number of ions available, this lower performance can be attributed to the low content of ions present in the electrolyte, and therefore, a lower specific capacitance is obtained. On the other hand, the thicker the electrolyte layer, the greater the distance between the electrolyte/electrode interface, which can result in a lower specific capacitance.^59^ For these reasons, the (1/2) configuration was selected for the construction of our subsequent devices. This optimized configuration was then used to characterize the influence of the PEDOT:PSS thickness, in the form of deposition time, on the performance of the supercapacitor devices. Other parameters such as the flow rate, working distance, and applied potential were also studied to optimize the deposition and performance of the devices (details in Supporting Information Figure S3). After this analysis, 60 μL h^–1^ flow rate, 7 cm working distance, and 15 kV applied voltage were chosen as the best performing values and used for further analysis (reaching 72 mF g^–1^ specific capacitance).

It is clear from the plots in Figure 7c,d that the deposition time has a strong influence on the capacitive performance of the devices with a visible increase in current density and hence in specific capacitance. The CV at different scan rates for a 30 min deposition is shown in Figure 7e. The cyclic voltammograms show a quasi-rectangular shape, with the background current increasing with the increase of PEDOT:PSS deposition time. However, current and specific capacitance increased for deposition times of 20–30 min. For lower times, the coating is not enough to add sites for charging the ions, while when the deposition time increases beyond 30 min (60 min), the specific capacitance decreases, which can be attributed to the increase in path length for electrolyte ions at the electrolyte/electrode interface. The higher specific capacitance of 72 mF g^–1^ was attributed to intercalation (ion diffusion in the pores of the composite electrode) of the gel-polymer composite electrode interface. For a 30 min deposition device, charge and discharge curves (GCD) were obtained at different current densities, and the plot of these curves is represented in Figure 7f. With this analysis, it is clear that for lower current densities (e.g., at 1.25 mA g^–1^), the charge and discharge times are larger. The curves present a triangular shape, suggesting an EDLC behavior, but for lower current densities, this triangular shape behavior starts to change, suggesting the presence of pseudocapacitive behavior.

**Figure 8:**
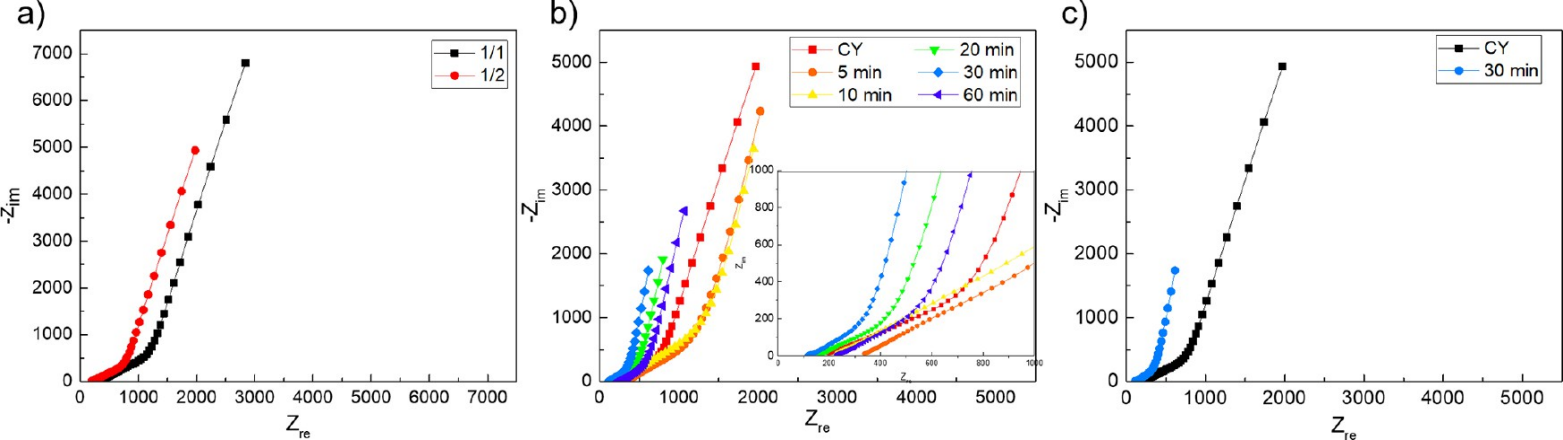
Electrochemical impedance spectroscopy: (a) comparison of different dip coatings of the electrolyte, (b) comparison of impedances of ESD wires for different time lengths, and (c) comparison of 30 min wire (best performing) with the pristine CY.

Electrochemical impedance spectroscopy (EIS) is an efficient method to measure the characteristic transient attributes of a supercapacitor device through the frequency response. The Nyquist plots for the CY with different dip-coating configurations and ESD PEDOT:PSS electrodes in symmetric two-electrode configurations were plotted, as shown in Figure 8, and the inset of the corresponding figures shows the magnified image of the plot in the high-frequency region. In the complex plane, the imaginary part (Zimag) denotes the capacitive property, and the real part (Zreal) represents the resistance attributes of the device. Traditionally, a Nyquist plot of the supercapacitor device comprises three distinct regions with respect to the frequency range. In the high-frequency range, device behavior corresponds to the interfacial charge transfer resistance of the electrode/electrolyte interface, and the low-frequency range is ascribed to the capacitive nature of the electrode active material.^60,61^ The medium frequency range is attributed to diffusion behavior that is an impact of the material porosities and surface states. The x-intercept of Zreal in the higher frequency range denotes the ohmic resistance (*R*_s_), which is comprised of the ionic resistance of the electrolyte, the intrinsic resistance of the active material, and the contact resistance at the interface of the active material and substrate (current collector). It is clear from Figure 8a that with the 1/2 configuration, *R*_s_ is lower than that of the 1/1 electrolyte configuration. It is also clear from the graph that the device fabricated with 30 min of ESD of PEDOT:PSS exhibited less *R*_s_ than the bare carbon yarn and other ESD. The Nyquist plot confirms its capacitive behavior in the high-frequency region and steeper slope in the low-frequency region. The absence of a characteristic semicircle in the Nyquist plot in the high-frequency region demonstrated low electronic/ionic resistance. The straight line in the low-frequency region is ascribed to the pure capacitive nature of the system owing to the fast transportation of charge carriers. The relatively low impedance of the 30 min ESD PEDOT:PSS electrode as compared to the pristine carbon yarn is attributed to the addition of PEDOT:PSS, which provides continuous channels through the porous network structure, which complement the electron/ion transport, as shown in Figure 8c. This also confirms the that strong adhesion between the electrode and electrolyte gel bequeaths the cell with low interface resistance.^62^

**Figure 9:**
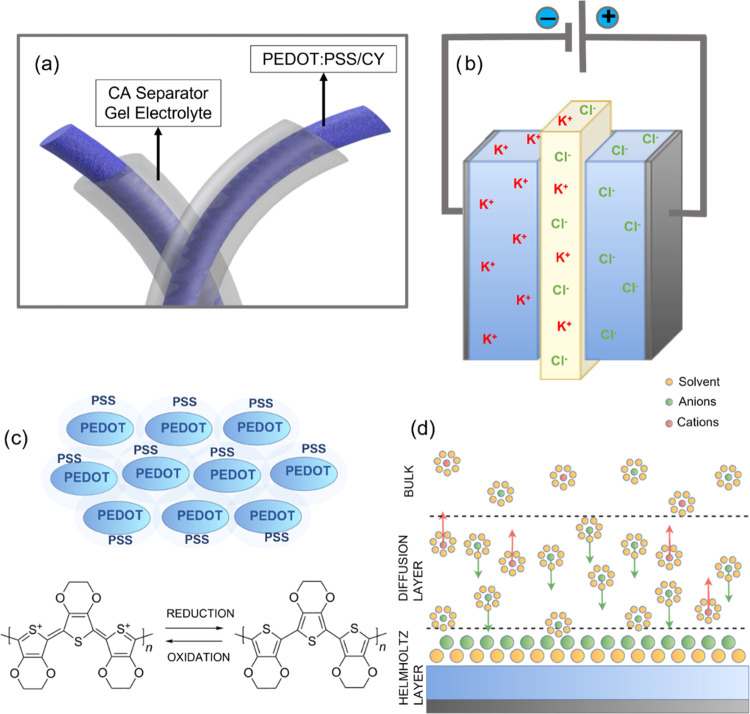
Graphical illustration and the charge storage mechanism of the PEDOT:PSS coating deposited on the carbon yarn. (a) Schematic of the PEDOT:PSS/CY device; (b) schematic of the device as a conventional capacitor with ion distribution; (c) schematic of PEDOT:PSS bonds and representation of oxidation states in the PEDOT:PSS bulk; and (d) schematic of electric double layer formation on the PEDO:PSS–electrolyte interface.

In Figure 9a,b, we see a representation of the device in the yarn, its transposition to a conventional device, and the ion distribution. Owing to the redox behavior in the conducting polymer and its conductivity, the device exhibited both EDLC and pseudocapacitance formation on the composite electrode surface, as shown in the schematic representation of Figure 9c,d. It is assumed that PEDOT:PSS has a two-phase structure (hole-conducting PEDOT and ion-conducting PSS grains), and both of them can contribute to the electrochemical reaction and hence to the total specific capacitance.^58^ The porous structure of PSS allows the diffusion of the electrolyte’s ions in the bulk of the structure, and the solvent in the electrolyte stimulates a screen effect between the cation PEDOT and anion PSS chain, which restricts the Coulombic interaction between them and facilitates the redox reaction.^63^ In addition to the solvent, the presence of PEG in the electrolyte also induces cracks in the separator matrix at the PEDOT:PSS interface, allowing for the electrolyte ions to penetrate the bulk of the active material and stimulate the redox reaction, enhancing the electrochemical performance of the material.^64^ Cations from the electrolyte (K^+^) enter the polymer channel, and oxidized cations of the polymer (PEDOT^+^) can be reduced to their pristine state by ion exchange with the electrolyte as follows (also demonstrated in Figure 9)
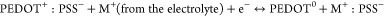
where M^+^ denotes the positively charged ion and e^–^ denotes the electron.

**Figure 10:**
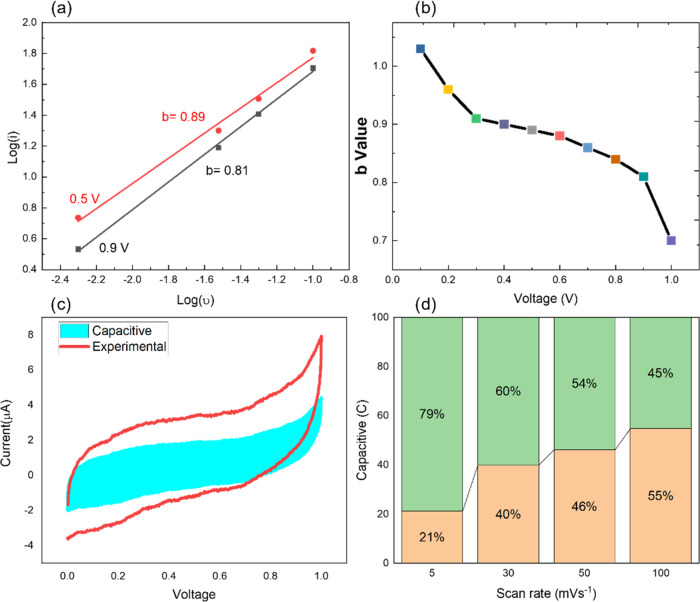
(a) *b*-Values as a function of the potential (0–1 V). (b) Power law dependence of current *i* on the scan rate υ shows good linearity. The two curves correspond to 0.5 V, *b* = 0.89 and 0.9 V, *b* = 0.81. (c, d) Percentage of the capacitive and diffusion-controlled contribution to experimental values at different scan rates.

The shape of the CV curve recorded at different scan rates details that a redox reaction occurs separately (oxidation on one electrode and reduction reaction on the other electrode).^65^ During electrochemical analysis, typical redox peaks were not clearly observed in low or high scan rates, as shown in Figure 7e. However, the quasi-rectangular shapes, especially at lower scan rates, denote the redox reaction in the PEDOT:PSS/CY composite electrode and are a typical case of pseudocapacitance behavior owing to a redox reaction in the conducting polymer.^66^ At lower scan rates, the electrolytes’ ions have enough time to diffuse in the bulk of the active material pores and exhibit pseudocapacitive behavior by exploiting all of the available active sites of the flexible composite electrode. On the other hand, at high scan rates, the device exhibited an ideal EDLC behavior and maintained a rectangular shape, indicating the fast surface charge storage of the device and hence high-power density. This is because at high scan rates, electrolyte ions only reach the surface of the electrode instead of the bulk of the material and hence reduce the number of active sites available for the electrolyte ions during the electrochemical reaction and intercalation. Generally, there are three different ways for charge storage in transition metal oxides or conducting polymers during the electrochemical process: (i) the diffusion-controlled faradaic contribution from the intercalation reaction; (ii) the faradaic contribution from the charge transfer with surface/surface atoms; and (iii) the ELDC effect (capacitive contribution).^67,68^ The quantitative analysis of the capacitive contributions can be performed by analyzing the CV recorded at different scan rates according to the power law
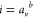
3In which, the current, *i*, obeys the power law with the scan rate, ν, and both *a* and *b* are adjustable parameters. When *b* approaches unity, it indicates that the charge stored is capacitive in nature. When *b* = 0, the current flow at any point of the potential is expected to vary with the square root of the scan rate, meaning that this is an ideal diffusion-controlled process. The *b*-value can be computed from the slope of the log(*i*) vs log(*v*) plots according to the above-mentioned equation. The calculated b-values for our PEDOT:PSS nanosphere film are detailed in Figure 10a. Figure 10b further exhibits two plots of log(*i*) vs log(*v*), indicating two different *b*-values of 0.89 and 0.81 at random potentials of 0.5 and 0.9 V, respectively. It is found during scanning from 0 to 1 V that the value of *b* is not always around 1, but it varies between 0.5 and 1. This implies that initially, the diffusional control process occurs, but at close to 0.98 V, it is predominantly capacitive control behavior with *b* = 0.75. This indicates that the PEDOT:PSS/CY device demonstrated both pseudocapacitive as well as ELDC behavior. To further quantify the diffusion control process of charge storage of the PEDOT:PSS nanosphere film, we separated the surface capacitive effects and diffusion-controlled intercalation contributions from the total charge storage as reported by Dun et al.^67^ The current response, *i*, by any material at a fixed voltage, *V*, can combine two distinct mechanisms, namely, the capacitive contribution *k*_1_υ (normally associated to faster kinetics) and the diffusion-controlled intercalation process *k*_2_ν^1/2^
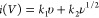
4

So, the above equation can be changed to
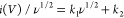
5According to eq 5, *k*_1_ and *k*_2_ constants can be computed from the slope and *y*-intercept of the graph of *i*(*V*)/ν^1/2^ vs ν^1/2^ as a straight line with the *y* = *mx* + b-type equation.

The *k*_1_ and *k*_2_ can quantify the capacitive and diffusion contributions of the current at fixed potentials. This allows determining the current resulting from the insertion of the K^+^ ions and that obtained from the capacitive effect and respective percentage contributions to the total stored charge. The capacitive and diffusion-controlled contributions to the total charge storage of the conducting polymer film electrode are demonstrated in Figure 10c,d. We are especially interested in the charge storage behavior of the PEDOT:PSS film at higher scan rates owing to the advantage of SCs over batteries is the ability to fast charge and high-power throughput. The deposition of thin-film coatings on flexible electrodes, however, exhibits a capacitive contribution of 0.58 mF at a high scan rate of 100 mV s^–1^ (short charging time of 10 s), which accounts for 55% of the total charge stored. There is still almost half of the contribution that comes from ion intercalation (diffusion-controlled). Moreover, as the scan rate decreases, the diffusion-controlled contribution becomes more prominent, with 80% to total charge storage. Although our calculation evidences the presence of pseudocapacitive behavior for the charge storage of PEDOT:PSS, the devices have exhibited excellent rate capability with capacitance decrease from 61 mF g^–1^ at 5 mV s^–1^ to 55 mF g^–1^ at 100 mV s^–1^.

**Figure 11:**
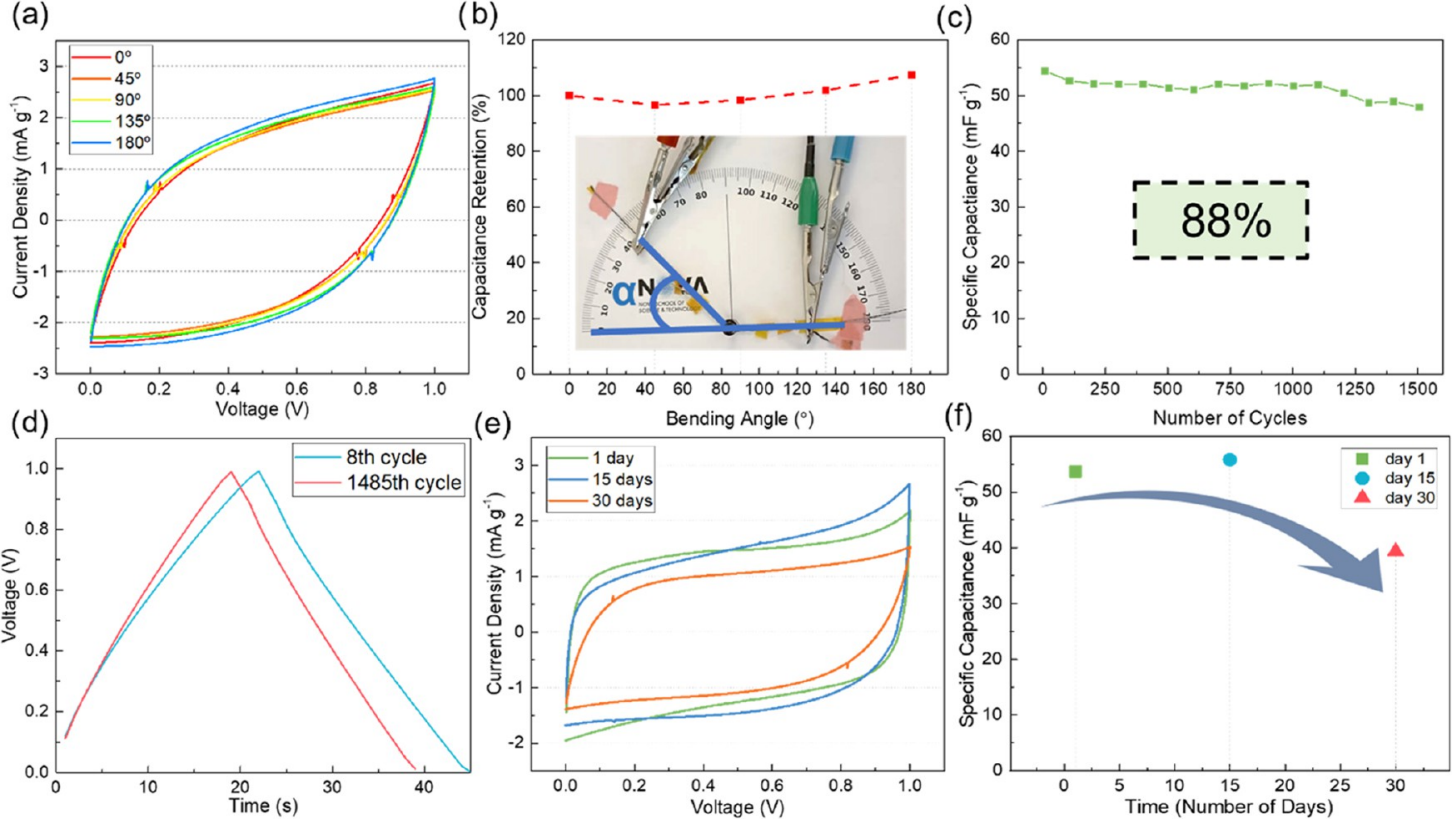
(a) Comparison of CV curves recorded at different bending angles; (b) comparison of specific capacitances at different bending angles; (c) graphical representation of cyclic stability; (d) comparison of cyclic stability GCD from the 8th and 1458th cycles; (e) comparison of the device stability test for 30 days; and (f) comparison of specific capacitance retention after 30 days.

In textile applications, electronic devices are subject to different mechanical stresses, and therefore, capacitors must withstand bending and twisting and be waterproof. The flexibility of the supercapacitors was tested by measuring the electrochemical properties with the device bent at different bending angles (0, 45, 90, 135, and 180°), showing excellent stability. The specific capacitance was calculated for the different bending angles, and a capacitance retention of 100% was obtained (refer to Figure 11a,b). In fact, there is an increase in capacitance for sharper bending angles (135 and 180°). Since the capacitor device was formed by winding two PEDOT:PSS/CY coated with the electrolyte, the contact points between the electrolyte are limited/weak, and therefore, bending promotes and forces the contact between the wires, increasing the ion transfer through the electrolyte, resulting in a higher specific capacitance. However, the present results demonstrated the suitability of the studied devices for integration in textiles due to high bending stability and capacitance retention. This also demonstrates that the fabricated devices can clearly withstand the mechanical stresses during the weaving process necessary for textile integration and during wearability.

The cyclic performance of the devices was evaluated by the galvanostatic constant charge and discharge for 1500 cycles at a constant current of 20 μA. The specific capacitance values were calculated from the discharge curve. The device has demonstrated remarkable cyclic stability with 88% capacitance retention after 1500 cycles, as shown in Figure 11c. The deterioration of the electrochemical performance of the device after 1500 cycles, as shown in Figure 11d, is attributed to the evaporation of the solvent in the electrolyte. The freshly fabricated devices have a higher content of electrolyte, and the devices have almost no voltage drop. This emphasizes the need for proper encapsulation of the devices, which prevents the evaporation of the electrolyte and hence ensures the long cyclic life of the devices.

The aging stability of the devices was also assessed over time, on the day of fabrication, 15 and 30 days after, as shown in Figure 11e,f. After 15 days of fabrication, no visible deterioration of performance was observed. After 30 days of fabrication, capacitance retention is 73% of the initial value. This degradation can be attributed to the evaporation of the electrolyte as devices were not encapsulated.

**Figure 12:**
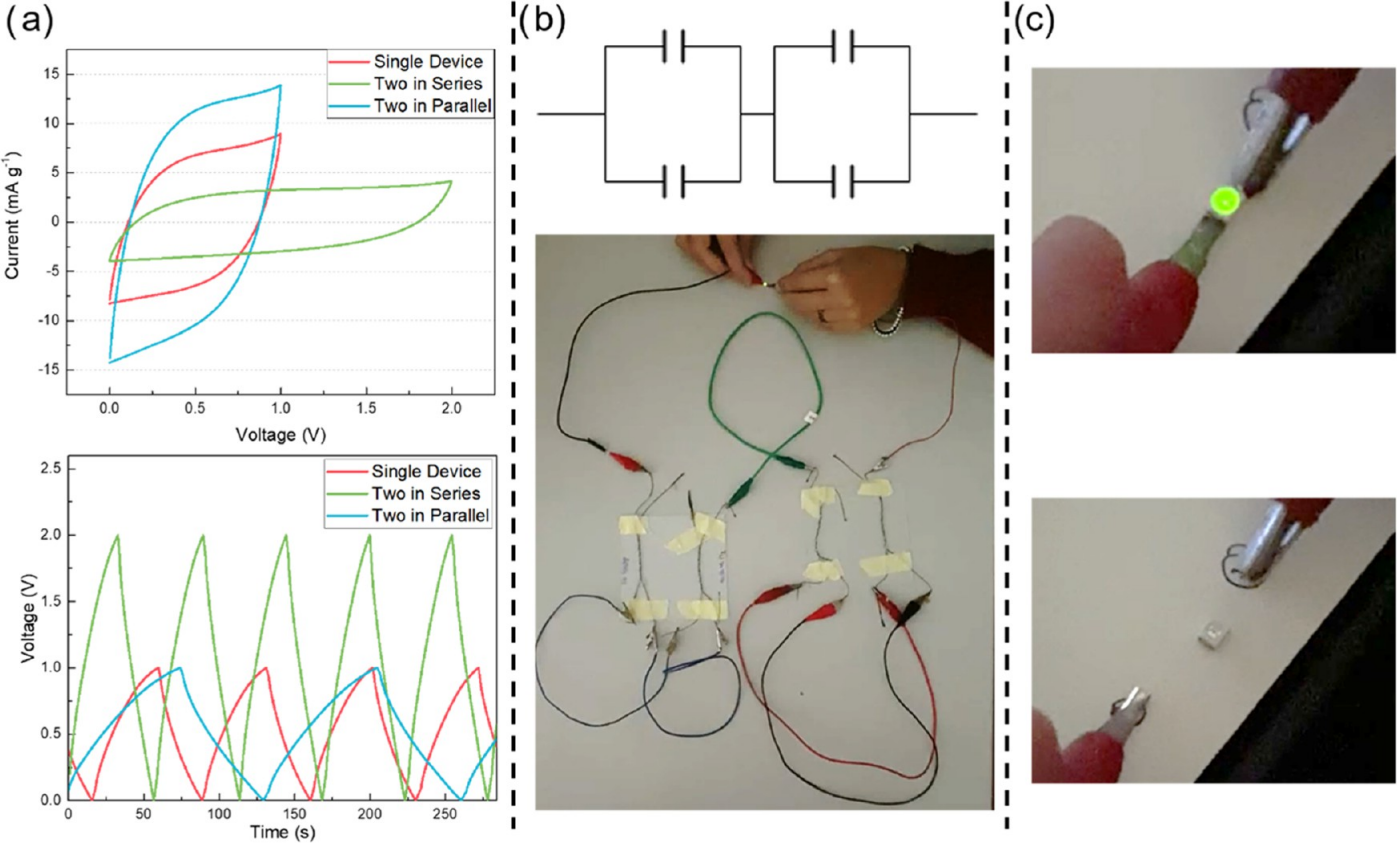
(a) Comparison of CV and charge/charge curves recoded for single devices and devices in series and parallel configurations; (b) schematic illustration of a circuit of two capacitors in parallel and in series with two others also in parallel, with a photograph of the circuit with the fabricated devices; and (c) two pictures of the LED lit by this circuit constructed with our PEDOT:PSS CA gel electrolyte devices.

The device connection in series or parallel is a requirement for power supply to electronic devices. As such, CV curves were recorded for two devices connected in parallel and in series at 100 mV s^–1^, as shown in Figure 12a. Doubling of the current is observed when connecting the devices in parallel, reaching 15 mA g^–1^, and doubling of the voltage when connecting them in series, increasing the voltage to 2 V, while both CV curves maintain their rectangular shape, meaning that no significant resistive losses have been introduced. Galvanostatic charge and discharge curves are also recorded in parallel and series configurations at a constant current of 1.25 mA g^–1^. The two series-connected supercapacitors were successfully charged to 2 V while maintaining a triangular shape of the charging and discharging curves to maintain the single device charge and discharge-like curve shape, confirming the lack of resistive losses introduced in the device’s behavior when integrated in a circuit. The discharging time for two parallel connected devices is higher than that of a single device at a potential ranging from 0 to 1 V, which is similar to what is reported in the literature.^69,70^ Connecting two devices in parallel and this subcircuit in series with another subsystem of two devices in parallel (as shown in Figure 12b) increases the circuit output voltage and discharge time. This configuration was used to light a light-emitting diode (LED) with a minimum operating voltage of 1.8 V (refer to Figure 12c). A video of the LED lighting, powered by the fabricated devices, can be found in the Supporting Information. The performance of the device was also compared with other PEDOT:PSS-based flexible devices, as shown in Table S2. The comparison shows the performance of the fabricated PEDOT:PSS-based fiber-shaped supercapacitors are comparable with other devices of the same PEDOT:PSS flexible supercapacitors class. Moreover, most of the literature reported devices have used acid-based gel-polymer electrolytes, and here, in this device, neutral salts are used during the preparation of the electrolyte.

## Conclusions

The possibility of coating carbon wires with PEDOT:PSS using the electrospray technique was demonstrated for the first time in this work. The PEDOT:PSS coating of the carbon yarns consists of nanoparticles that agglomerate and form a porous and conductive coating which is very suitable for charge storage. Hence, PEDOT:PSS/CY was used in the fabrication of symmetrical capacitors, in which two PEDOT:PSS/CY with a cellulose-based gel electrolyte were interwoven together. The interlacing of the two yarns allows for only a very reduced contact through the electrolyte of both electrodes and still, with this reduced area, the device shows a specific capacitance of 72 mF g^–1^, over 85% retention after 1500 charge–discharge cycles, and 100% retention when bending the wire completely over itself (at 180°). Furthermore, the produced devices were shown to have a pseudo-capacitor behavior with a high electrical double-layer contribution, as the PEDOT:PSS coating contributes to oxidation/reduction reactions, and the inherent porosity allows the electrolyte to penetrate the surface area of the electrode. Data post-processing has shown that both diffusion and capacitive effects contribute to the total charge storage, although with different percentages depending on the scanning rate. A series–parallel circuit with 4 devices showed a good linearity of current and voltage values so that the resistance effect was minimal. This circuit proved to generate enough power for an LED and make it shine; hence, metrics imply the use of more devices in such a higher energy-consuming circuit. Thus, this work represents a significant step forward in the development of electrospray deposition as a viable and promising technique for fiber functionalization. Also, this work shows a development in the field of flexible textile devices for energy storage and supply and has the potential to catalyze further opportunities for the advancement of flexible electronic circuits in textile applications.

## Supporting Information Available

Electrospray deposition setup image (Figure S1); PEDOT:PSS main Raman vibrational modes (Table S1); optical microscope images of the electrolyte over carbon yarns (Figure S2); electrochemical analysis of EDS parameters (Figure S3); comparison of PEDOT:PSS-based fiber-shaped supercapacitors found in literature with this work (Table S2) (PDF)
LED being lit with the use of fabricated devices (MP4)
