# Supplementary material for: Electrospray Deposition of PEDOT:PSS on Carbon Yarn Electrodes for Solid-State Flexible Supercapacitors
Source: ACS Appl Mater Interfaces. 2023 Jun 19;15(25):30727–41. doi: 10.1021/acsami.3c03903 (PMC10316332; doi:10.1021/acsami.3c03903)
Supplement: Supplementary file 1 — am3c03903_si_001.pdf [file am3c03903_si_001.pdf]

SUPPORTING INFORMATION

Electrospray Deposition of PEDOT:PSS on Carbon Yarn Electrodes for Solid-State Flexible Supercapacitors

Authors: Mariana P. Moniz[1] *, Amjid Rafique[1], João Carmo[1], J. P. Oliveira[1], Ana Marques[1,2], Isabel M. M. Ferreira[1] and Ana Catarina Baptista[1] *

[1]CENIMAT|i3N, Department of Materials Science, School of Science and Technology, NOVA University Lisbon, 2829-516 Caparica, Portugal

[2] Physics Department, Faculty of Sciences, University of Lisbon, 1749-016 Lisbon, Portugal

***Corresponding authors: anacbaptista@fct.unl.pt (AC Baptista) and m.moniz@campus.fct.unl.pt (MP Moniz))**

Figure S1 shows the ESD setup.

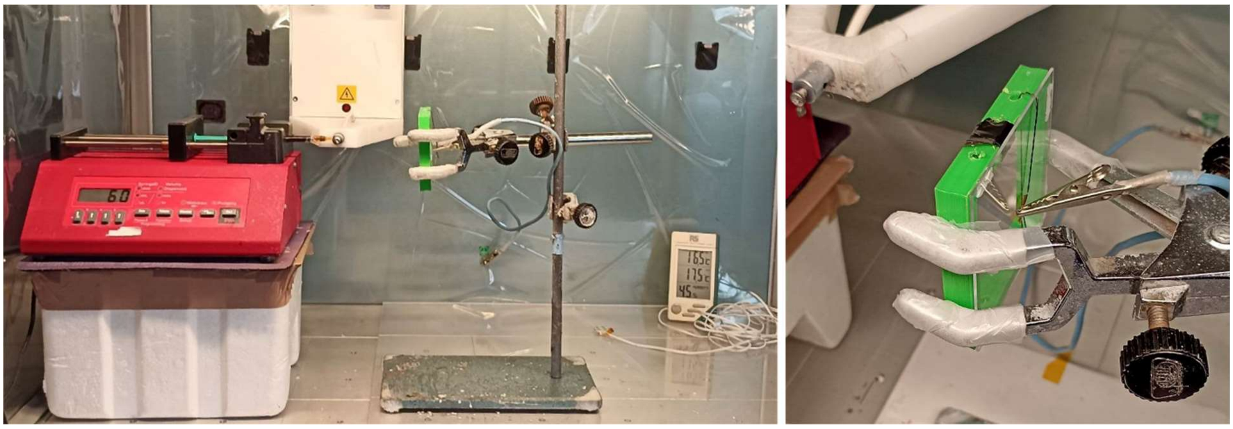

Figure S1. On the right an image the whole ESD setup; On the left a closeup of the supporting frame for the CY.

Table S1 lists the main vibrational modes identified in the measured PEDOT:PSS Raman spectra, which agrees with the literature [1, 2]

Table S1. PEDOT:PSS main Raman vibrational modes under 532 nm excitation laser wavelength.

Vibration peak (cm^{-1})	Description
437	PEDOT: C–O–C deformation
529	
575	Oxyethylene ring deformation
697	PEDOT: C–O–C deformation
852	PEDOT: C–S–C deformation
987	PSS: Oxyethylene ring deformation
1099	PSS: stretching modes of SO_3^-
1121	
PEDOT THIOPHENE RINGS VIBRATION MODES	
1252	$C_\alpha - C_{\alpha'}$ inter-ring stretching
1368	$C_\beta - C_{\beta'}$ stretching deformations
1438	$C_\alpha = C_\beta$ symmetrical stretching
1499	$C_\alpha = C_\beta$ asymmetric stretching
1532	$C_{\alpha'} = C_{\beta'}$ asymmetric stretching
1568	$C_\alpha = C_\beta$ asymmetric stretching

Figure S2 depicts the OM images used to estimate the thickness of the electrolyte dips. Figure S2 detailed the three OM images of pristine CY, CY with 1 dip of electrolyte, and CY with 2 dips of electrolyte. For each micrograph at least 3 measurements of the wire thickness were performed.

The average thickness and standard deviation were calculated for each sample: CY is 443.70 ± 53.36 µm thick, the CY with 1 dip of electrolyte is 540.31 ± 41.25 µm thick and the CY with 2 dips of electrolyte is 739.74 ± 83.03 µm thick. To get the thickness of just the electrolyte, the thickness of just the electrolyte layer, we subtracted the thickness of the CY to the other two value, arriving to a thickness of 1 dip of electrolyte of 96.61 ± 67.44 µm, and 2 dips of electrolyte, 296.04 ± 98.70 µm. The high standard deviation values can be attributed to the coiled configuration of the yarn, which contributes to the variation in the thickness of the electrolyte coating, especially for the thinner electrolyte layer.

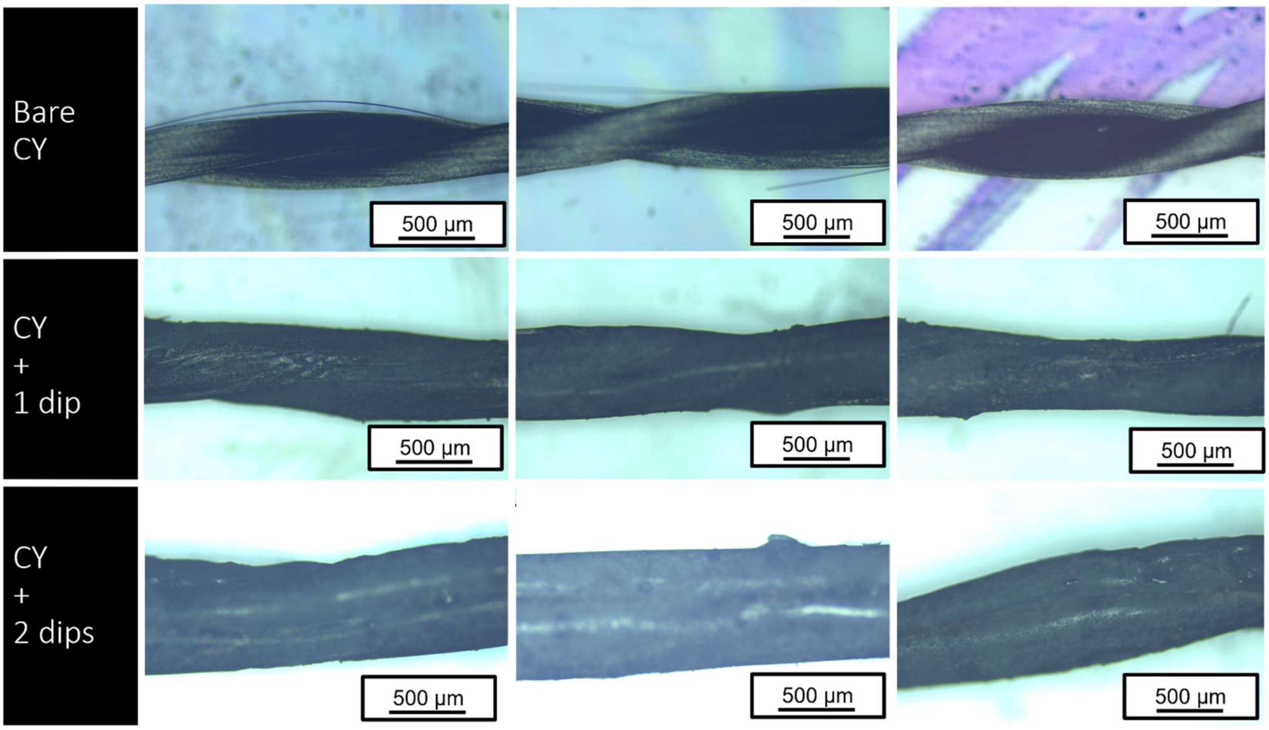

Figure S2. OM images of bare CY, of CY with 1 dip of electrolyte and CY with 2 dips of electrolyte.

A further study was made on the electrochemical performance of the devices assembled with PEDOT:PSS coated electrodes produced under several ESD parameters. The influence on the electrochemical behaviour of variations in flow rate, working distance and applied voltage was analysed. From Figure S3 a) it is noticeable the influence of the flow rate in the electrochemical performance of the devices. Considering the time window, the 40 μLh^{-1} flow rate probably leads to a lack of sufficient deposition to significantly improve the electrochemical performance of the devices. Also, for the 80 μLh^{-1} it is possible to observe that it has lower specific capacitance than the one obtained for 60 μLh^{-1}. This can be attributed to over deposition of material, creating a layer of PEDOT: PSS that is too thick or due to an increase in particle size, which can occur with an increase in flow rate [3]. In Figure S3 b), the differences of electrochemical performance obtained for different working distances are not so pronounced. It is noticeable that a higher performance is obtained for a working distance of 7 cm, but contrary to what expected from the morphological analysis, the obtained performance of 4 cm deposition distance is higher when compared with the 10 cm working distance. This better performance of the 4 cm working distance might be caused due to a more uniform and continuous deposition, increasing the conductivity of the PEDOT:PSS layer, but lacking the effect of high surface area obtained for 7 cm, where a film of individualized particles is formed. In Figure S3 c) the variation of

electrochemical performance of applied voltage is shown. There is an evident difference from 18 kV to the depositions of 12.4 kV and 15 kV, which have rather similar electrochemical performance. Clearly 18 kV is a too high working voltage to perform this deposition, maintaining the distance at 7 cm and flow rate at 60 μLh^{-1}. Between the depositions performed at 12.4 kV and 15 kV, the difference is less acute. For 12.4 kV at 5 mVs^{-1} a specific capacitance of 82 mFg^{-1} is reached and for 15 kV of 72 mFg^{-1}, but at 100 mVs^{-1} the opposite happens, where for 12.4 kV a specific capacitance of 53 mFg^{-1} is achieved and at 15 kV, a specific capacitance of 62 mFg^{-1}. With 15 kV the obtained PEDOT:PSS deposition was morphologically more uniform and for that reason was selected as optimal voltage condition, but in terms of electrochemical behaviour this parameter variation does not have a major impact. Regarding this study the deposition with 60 μLh^{-1} flow rate, 7 cm working distance, and 15 kV applied voltage were chosen as the best performing.

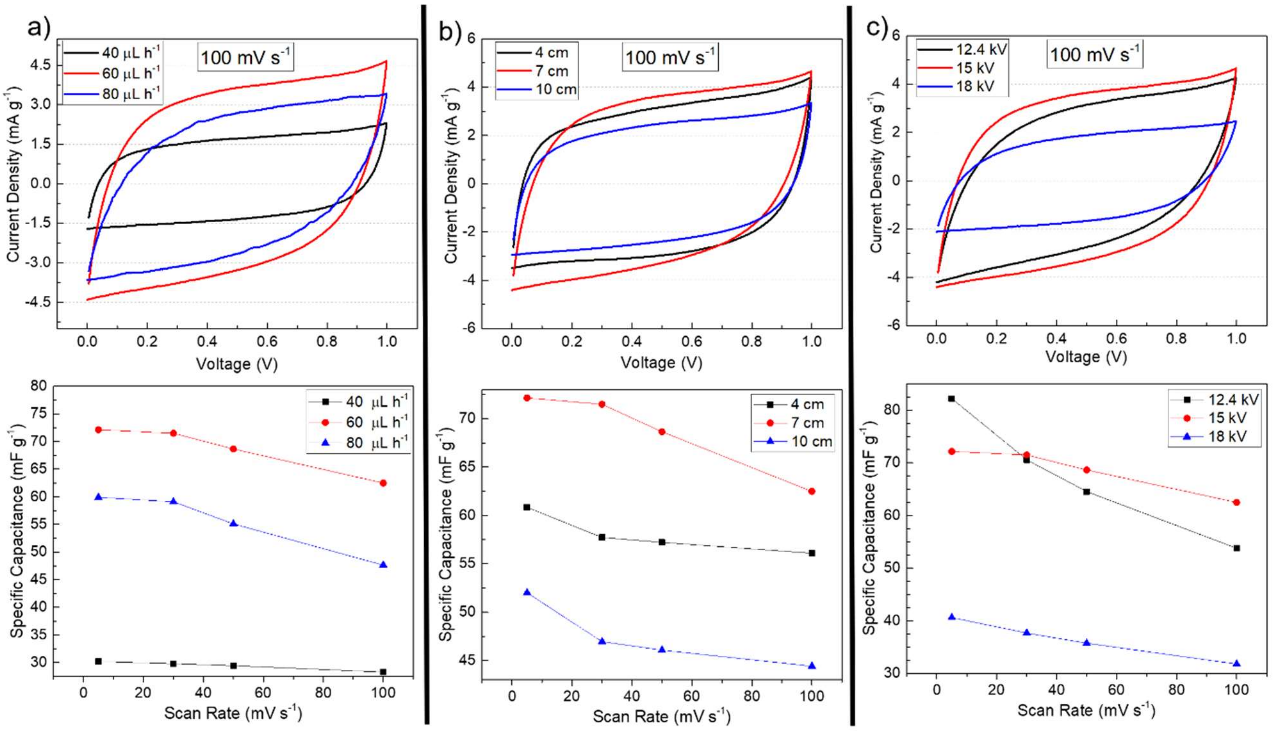

Figure S3. Electrochemical analysis of ESD parameters; a) Electrochemical analysis of flowrate; b) Electrochemical analysis of working distance; c) Electrochemical analysis of Applied Voltage.

Table S2 was constructed to make the comparison of PEDOT:PSS based flexible fiber shaped supercapacitors. The comparison shows that our PEDOT:PSS based flexible fiber shaped supercapacitors exhibited an excellent performance as compared to other devices of the same flexible supercapacitor's class.

For this comparison, the calculation of specific capacitance (SC) using just the mass of PEDOT:PSS, independent of the weight of the CY, was calculated using a high precision scale, and the measured weight was of 0.142 ± 0.018 mg. Also, the calculation of the surface area of one electrode, assuming the shape of this electrode as an elliptic cylinder was calculated, using the measured values for the thickness of the yarn, on equation S1 (an approximation of the calculation of the perimeter of an ellipse).

$$P = 2\pi \cdot \sqrt{\frac{a^2 + b^2}{2}} \qquad (S1)$$

Where *a* is the radius of the smaller axis of the ellipse and *b* the radius of the biggest axis of the ellipse. With this calculation, multiplying by the active length of the device (6 cm) we found a surface area for the yarn of 0.842 cm^2.

Table S2. Comparison table of relevant similar work, with analysis of active material, electrolyte, substrate material, working voltage (W. V.), separator material, specific capacitance (SC) in Fg^{-1}, SC in $mFcm^{-1}$ and SC in $mFcm^{-2}$.

#	Active Material	Electrolyte	Substrate	W. V.	Separator	SC (Fg^{-1})	SC ($mFcm^{-2}$)	Ref.
2	PEDOT:PSS	Artificial Sweat Solution	Polyester/ cellulose	1.3	Polyester/ Cellulose	5.5	10	[4]
3	PEDOT:PSS@ KNF	PVA/ H_2PO_4	Kevlar Fabric	0.6	PVA/H_2PO_4	2.4		[5]
4	PEDOT:PSS	Artificial Sweat Solution	cellulose/ polyester cloth	0.8	Cellulose fibers	2.9		[6]
5	PEDOT:PSS fibers	PVA/ H_2PO_4	PEDOT:PSS yarn	1	PVA/H_2PO_4		115	[7]
6	IRCNT@ PEDOT:PSS	PVA/ H_3PO_4	CNT yarn	1		18.5		[8]
7	CC/ PEDOT: PSS	PVA/ H_3PO_4	Carbon cloth	1	PVA/ H_3PO_4	73		[9]
8	PEDOT:PSS/ AgNFs	PVA/ H_2PO_4	Transparent film	1	PVA/H_2PO_4		0.91	[10]
9	PEDOT-coated polyester fabric	PVA/ H_2PO_4	PEDOT-coated polyester fabric	1	PVA/ H_2PO_4		0.64	[11]
10	PEDOT:PSS/ SWCNT	hydroxyethy l cellulose-potassium chloride	Jute fibers	0.8	cellulose-based material		8.65	[12]
11	PEDOT: PSS/ CY	Cellulose acetate gel-electrolyte	CY	1	Cellulose acetate gel-electrolyte	0.072* 4.27**	0.72	This work

* Calculated using the full mass of the electrode (CY + PEDOT:PSS).

** Calculated using only the mass of PEDOT:PSS

References

1. Chen, Y., et al. *Enhanced electrochemical performance of PEDOT film incorporating PEDOT: PSS*. in *2015 2nd International Conference on Machinery, Materials Engineering, Chemical Engineering and Biotechnology*. 2015. Atlantis Press.
2. Farah, A.A., et al., *Conductivity enhancement of poly (3, 4-ethylenedioxythiophene)-poly (styrenesulfonate) films post-spincasting.* Journal of Applied Physics, 2012. **112**(11): p. 113709.
3. Bock, N., et al., *Electrospraying, a reproducible method for production of polymeric microspheres for biomedical applications.* Polymers, 2011. **3**(1): p. 131-149.

4. Manjakkal, L., et al., *A wearable supercapacitor based on conductive PEDOT: PSS-coated cloth and a sweat electrolyte.* Advanced Materials, 2020. **32**(24): p. 1907254.
5. Gibertini, E. and L. Magagnin, *PEDOTS: PSS@ KNF Wire-Shaped Electrodes for Textile Symmetrical Capacitor.* Advanced Materials Interfaces, 2022. **9**(20): p. 2200513.
6. Manjakkal, L., et al. *Flexible Supercapacitor with Sweat Equivalent Electrolyte for Safe and Ecofriendly Energy Storage*. in *2020 IEEE International Conference on Flexible and Printable Sensors and Systems (FLEPS).* 2020. IEEE.
7. Yuan, D., et al., *Twisted yarns for fiber-shaped supercapacitors based on wetspun PEDOT: PSS fibers from aqueous coagulation.* Journal of Materials Chemistry A, 2016. **4**(30): p. 11616-11624.
8. Su, F. and M. Miao, *Flexible, high performance two-ply yarn supercapacitors based on irradiated carbon nanotube yarn and PEDOT/PSS.* Electrochimica Acta, 2014. **127**: p. 433-438.
9. Kumar, N., R.T. Ginting, and J.-W. Kang, *Flexible, large-area, all-solid-state supercapacitors using spray deposited PEDOT: PSS/reduced-graphene oxide.* Electrochimica Acta, 2018. **270**: p. 37-47.
10. Singh, S.B., et al., *Embedded PEDOT: PSS/AgNFs network flexible transparent electrode for solid-state supercapacitor.* Chemical Engineering Journal, 2019. **359**: p. 197-207.
11. Yu, X., et al., *Stretchable, Conductive, and Stable PEDOT-Modified Textiles through a Novel In Situ Polymerization Process for Stretchable Supercapacitors.* Advanced Materials Technologies, 2016. **1**(2): p. 1600009.
12. Manjakkal, L., et al., *Natural Jute Fibre-Based Supercapacitors and Sensors for Eco-Friendly Energy Autonomous Systems.* Advanced Sustainable Systems, 2021. **5**(3): p. 2000286.
